# Qualitative Differences in Attribution of Mental States to Other People in Autism and Schizophrenia: What are the Tools for Differential Diagnosis?

**DOI:** 10.1007/s10803-021-05035-3

**Published:** 2021-04-28

**Authors:** Monica Mazza, Maria Chiara Pino, Roberto Keller, Roberto Vagnetti, Margherita Attanasio, Angela Filocamo, Ilenia Le Donne, Francesco Masedu, Marco Valenti

**Affiliations:** 1 Department of Applied Clinical Sciences and Biotechnology, University of L’Aquila, Via Vetoio, Località Coppito, 67100 L’Aquila, Italy; 2 Reference Centre for Autism of the Abruzzo Region, Local Health Unit ASL 1, L’Aquila, Italy; 3 Reference Centre for Adult Autism of the Piemonte Region, DSM Local Health Unit ASL Città di Torino, Turin, Italy

**Keywords:** Autism spectrum disorder (ASD), Schizophrenia spectrum disorders (SSD), Theory of Mind (ToM), Differential diagnosis

## Abstract

The differential diagnosis between schizophrenia spectrum disorders (SSD) and autism spectrum disorders (ASD) remains an important clinical question, because they have overlap in clinical diagnosis. This study explored the differences between ASD (n = 44) and SSD patients (n = 59), compared to typically developing peers (n = 63), in completing an advanced Theory of Mind (ToM) task. The outcome found several differences between groups. The SSD patients showed greater difficulty in understanding social scenarios, while ASD individuals understood the stories, but did not correctly identify the protagonist’s intention. The interesting aspect of the results is that some ToM stories are more informative about the mentalistic reasoning of the two clinical groups, namely, the stories that investigate pretend, persuasion, double bluff and ironic joke constructs.

**Supplementary Information:**

The online version contains supplementary material available at 10.1007/s10803-021-05035-3.

## Introduction

The relationship between autism spectrum disorders (ASD) and psychosis, especially schizophrenia, is complex and poorly studied in the literature. ASD are neurodevelopmental disorders characterised by a deficit in social communication and interaction and by restricted, repetitive patterns of behaviour, interests or activities (American Psychiatric Association, 2013; Lai et al., 2014). The prevalence of ASD in the general population is estimated to be around 1% worldwide, based on both screening and register-based studies (Valenti et al., 2019). Usually, symptomatology appears during early childhood and has a lifespan impact. Schizophrenia spectrum disorders (SSD) frequently manifest in adolescence or young adulthood and are characterised by delusions, hallucinations, disorganised thinking and speaking, grossly disorganised or abnormal motor behaviour and negative symptoms (American Psychiatric Association, 2013). Estimates of the international prevalence of schizophrenia among non-institutionalised persons range between 0.30 and 0.75% (Moreno-Küstner et al., 2018). The term ‘spectrum’ refers to the various manifestations of both disorders, and accounts for the complexity of symptomatology that varies according to the level of severity and manifestation of the symptoms. This complexity can mislead the clinician when he/she engaged in the differential diagnostic process.

According to the literature, ASD and SSD share clinical, neurobiological and cognitive features (Barlati et al., 2016; Bechi et al., 2020; Bertelli et al., 2015; Chandrasekhar et al., 2020; Deste et al., 2020; King & Lord, 2010; Sasson et al., 2011), and this makes distinguishing between the two disorders particularly difficult, especially in adolescents or young adults when they arrive at clinical services without any diagnosis.

Although the first diagnosis of ASD is usually made in childhood, it is not uncommon in adulthood.

Many individuals with ASD have above or average cognitive abilities and are able to use compensatory mechanisms or, in some case, live in environments with an adequate support system.

Moreover, ASD symptoms may be masked by the co-occurrence of other neurodevelopmental or neurological disorders (Bastiaansen et al., 2011; Kan et al., 2008; Wing & Potter, 2002). ASD individuals with an average to above average cognitive abilities are more likely to receive an erroneous diagnosis of psychosis (Keller et al., 2015; Larson et al., 2017; Luciano et al., 2014; Tin et al., 2018), particularly when the clinical history is not clear and positive symptoms (i.e. delusions or hallucinations) of SSD are not very explicit (Tin et al., 2018).

### Clinical Assessment and Misdiagnosis

Some symptoms in both disorders have common features, and the clinician is not always able to refer these symptoms easily to one or the other disorder. For example, social difficulties and pragmatic and grammatical language impairment are characteristics of both conditions (Chisholm et al., 2015; De Crescenzo et al., 2019; Solomon et al., 2011). Language deficits typical of autism appear similar to those found in prodromal symptoms of schizophrenia (De Crescenzo et al., 2019; Owen et al., 2011). In addition, formal thought disorder is similar to tangential thought, formal language, and the focus on restrictive interests shown by people with autism. Likewise, a lack of verbal initiation and poverty of speech content seems to be common in both disorders (De Crescenzo et al., 2019; Solomon et al., 2011).

Even the use of clinical tests for the diagnosis of ASD does not always help the clinician in the differential diagnostic process. Moreover, diagnostic tools designed for adulthood are limited (Maddox et al., 2017).

The standard diagnostic test for autism is the Autism Diagnostic Observation Schedule—Version 2 (ADOS-2; Lord et al., 2012), but despite its widespread use, it is worth noting that ADOS-2 is not specific enough when used in adolescents or adults with psychotic onset (Frith & Happé, 2005; Keller & Bari, 2019; Pino et al., 2018). Social withdrawal and social communication difficulties in ASD usually overlap with negative SSD symptoms or catatonic behaviour, thereby leading to diagnostic errors (Bastiaansen et al., 2011; Frith & Happé, 2005). Several studies have shown that ADOS-2 Module 4, usually intended for adolescents and adults with fluent language skills, might not correctly distinguish between ASD and psychosis, producing a high rate of false positive (Bastiaansen et al., 2011; de Bildt et al., 2016; Hus & Lord, 2014; Maddox et al., 2017; Mazza et al., 2010). Indeed, some items in the Communication (conversation, emphatic or emotional gestures) and Reciprocal Social Interaction domains of ADOS-2 Module 4 (unusual eye contact, facial expression directed towards others, empathy towards/comments on others’ emotions, quality of social overtures, quality of social responses, amount of social reciprocal communication) could reflect negative symptoms of psychosis rather than typical features of ASD.

A tool that could help the clinician during the diagnostic process is the Autism Diagnostic Interview—Revised (ADI-R; Lord et al., 1994), a semi-structured interview administered to caregivers in order to collects developmental information on the patient’s early life. However, ADI-R could lead to false negative in Asperger’s syndrome because symptoms are detected later, upon entering school; moreover, use with adult patients may be limited by the age of the parents and the quantity and quality of memories relating to the child’s early life and developmental milestones (Keller & Bari, 2019).

Based on recent literature and on diagnostic criteria of DSM-5 (American Psychiatric Association, 2013), the problem of a correct diagnosis, especially when the clinician faces an adolescent or adult with social withdrawal, anxiety, compulsions and/or stereotyped thinking, remains a very important clinical question.

### Neuroimaging Prospectives

The relationship between ASD and SSD is consistent with a neurobiological model that suggests a common basis for ASD and SSD, with several genetic alterations or neuroimaging investigation (Biamino et al., 2016; de Lacy & King, 2013; King & Lord, 2010). Neuroimaging studies showed common abnormalities in gray matter volumes and brain activations (Cheung et al., 2010; Pinkham et al., 2008; Sugranyes et al., 2011; Yoshihara et al., 2020). Areas with the greatest overlap are those correlated with Social Cognition (SC). SC is a complex set of cognitive abilities involved in the processing and interpretation of social stimuli (Bishop-Fitzpatrick et al., 2017; Mazza et al., 2010, 2017; Pino et al., 2017, 2020). Sugranyes et al. (2011) suggested that both ASD and SSD were characterised by media-temporal and ventrolateral prefrontal hypoactivity during SC tasks. Similarly, Pinkham et al. (2008) found reduced brain activity in the amygdala, fusiform gyrus and ventrolateral prefrontal cortex in individuals with autism and paranoid schizophrenia during trustworthiness of human faces tasks (Eack et al., 2017). Comparable reductions in superior temporal sulcus and ventromedial prefrontal cortex activity were found by Ciaramidaro et al. (2015) during a Theory of Mind (ToM) task. ToM is a main component of SC which can be summarised as the ability to understand mental and emotional states of other people (Mazza et al., 2014, 2017; Pino et al., 2020).

From a neuroimaging perspective, a voxel-based meta-analysis observed an overlap between clusters of brain alteration in ASD and SSD, involving areas related to the sense of the self, interoception, emotions and SC (Cauda et al., 2017). Interestingly, in ASD and SSD, brain alterations do not distribute randomly but rather follow network-like patterns of co-alterations, involving highly connected nodes.

### Theory of Mind Abilities

Deficits in ToM abilities are considered mechanisms underlying social dysfunctions of both disorders. On the other hand, differences in ToM between ASD and SSD are delineated during specific phases of development; indeed, Frith (1995, 2004) suggests that, in contrast to ASD individuals, ToM abilities in people with psychosis develop normally but become impaired following the first psychotic episode (Bliksted et al., 2017; Mazza et al., 2001). People with psychosis may lose an ability they once had (Mazza et al., 2001), and that makes them socially similar to people with ASD.

However, the literature provides opposing evidence about the hypothesis that ToM is similarly compromised in both disorders (Chung et al., 2014; Deste et al., 2020; Fernandes et al., 2018; Morrison et al., 2017; Pinkham et al., 2019; Sasson et al., 2011; Tin et al.,, 2018) and that it could be disorder-specific (Brighenti et al., 2018; Colle et al., 2019). According to Morrison et al. (2017), the two disorders may differ in the nature and mechanisms of ToM deficit, but these differences could remain unidentified due to a lack of direct comparisons between ASD and SSD via systematically matched designs.

Some studies (Bowler, 1992; Couture et al., 2010; Craig et al., 2004; Murphy, 2006; Pilowsky et al., 2000) found that ASD and SSD patients did not differ in ToM verbal and visual tasks.

Tin et al. (2018) showed that ASD individuals, when directly compared with SSD patients, performed poorer in verbal but not in visual ToM. The authors conclude that ASD individuals shared similar but more severe ToM impairments than SSD patients did.

Martinez et al. (2019) showed that individuals with SSD were significantly impaired in the attribution of intentions to others (ToM), and that they did not differ from persons with ASD in the animated shapes task.

According to Frith (1992), social difficulties in ASD are correlated with poor mentalising ability; in particular, individuals with ASD are unable to represent the mental states of other people (Frith, 1992; Pickup & Frith, 2001; Tin et al., 2018). On the contrary, hypermentalising and an erroneous representation of others’ mental states are typical of SSD (Frith, 1992; Pickup & Frith, 2001; Tin et al., 2018). In line with what Frith claimed in 1992, many studies have suggested that social impairments in the two disorders may manifest in different ways: ASD are characterised by ‘*under-mentalising*’ and SSD tends towards ‘*over-mentalising*’ in ToM tests. Indeed, SSD patients have a higher degree of intentionality in their interpretations (Bliksted et al., 2016; Crespi & Badcock, 2008; Crespi et al., 2010; Martinez et al., 2019; Morrison et al., 2017). Fernandes et al. (2018) highlight that the unclear results reported in the literature about differences between ASD and SSD in terms of ToM performance could be related to a lack of tools able to discriminate between the disorders. The difficulty of researchers and clinicians in understanding the different performance of the two groups in ToM tests may be due to the lack of sensitivity of the tests used (Baksh et al., 2020; Brewer et al., 2017; Kuo et al., 2019; Morrison et al., 2019; Pino et al., 2020). Moreover, the development of ToM test for ASD individuals reports limitations, such as the fact that ToM tasks are performed in experimental settings that are different from the social situations of real life.

Hutchins et al. (2012, 2016) highlighted that ToM tasks require explicit and applied skills. The authors define explicit ToM competence as conceptual and logical knowledge that is limited to experimental contexts. Instead, applied ToM competence would be characterised by the ability to apply ToM knowledge to address social situations presented by the real world (Hutchins et al., 2016). Explicit and applied ToM competencies may be dissociated; for example, although adults with Asperger’s syndrome perform explicit tasks, they may use logical rather than mentalistic processes to provide the correct response (Bowler, 1992; Hutchins et al., 2016).

In addition, several ToM tests are based on language abilities. According to the literature, ToM skills and development of language are related (Apperly et al., 2009; Astington & Jenkins, 1999; Milligan et al., 2007; Pino et al., 2018; Sivaratnam et al., 2012). Several studies highlighted that individuals with ASD and SSD exhibit atypical cognitive, language and social functioning (Mazza et al., 2008; Solomon et al., 2011). Moreover, some studies showed that deficit in ToM abilities may be associated with sematic-pragmatic language, which is compromised both in ASD and SSD (Bambini et al., 2016; Deliens et al., 2018; Mazza et al., 2008; Pawelczyk et al., 2020).

### Aim of Study

There is enough evidence, since the pioneer research of Happé (1994), to hypothesise that individuals can use two ways to understanding the intention of others: a ‘mental’ reasoning and a ‘physical’ reasoning. The first involves mental states because it refers to feelings, thoughts, desires, dispositions and traits. The second is based on non-mental events such as the action of objects, physical events, physical appearances and outcomes.

Based on these assumptions, the aim of our study is: (a) to evaluate and describe differences in the style of reasoning used to solve Advanced ToM tasks (Blair & Cipolotti, 2000; Happé, 1994) between typically developing (TD) individuals and clinical populations (ASD and SSD); and (b) to understand whether the reasoning style used for the mentalising task differs between ASD and SSD. From a translational perspective, the overall goal is to offer clinicians an efficient way to identify items of the ToM test that discriminate between ASD and SSD.

## Methods

### Participants

One hundred and sixty-six subjects participated in this study, classified in three groups:

- 63 undergraduate students (42 males and 21 females) attending the Master’s degree course in psychology at the University of L’Aquila, Italy, with a mean age of 21.5 years (standard deviation (SD) 3.02 years). The exclusion criteria for the recruitment was a history of neurological disease, including epilepsy, psychiatric disorders, substance disorders, head trauma and cognitive impairment.
- 44 individuals with level-1 ASD, according to DSM-5 criteria (American Psychiatric Association, 2013) (36 males and 8 females), with a mean age of 21.9 years (SD 6.92), recruited by the Reference Regional Centre for Autism (CRRA) in L’Aquila, Italy. ASD diagnoses were made by experienced psychiatrists and psychologists according to the DSM-5 criteria (American Psychiatric Association, 2013) and using the ADOS-2 Module 4 (Lord et al., 2012). Exclusion criteria were cognitive impairment, epilepsy and language disorders.
- 59 subjects affected by SSD (43 males, 16 females), with a mean age of 21.9 years (SD 2.31), coming for treatment due to First Episode Psychosis at the Laboratory of Clinical Neuropsychology of the Department of Applied Clinical Sciences and Biotechnology, University of L’Aquila, Italy. According to medical records and clinical interviews conducted by experienced psychiatrists with patients and their parents, none of the SSD individuals showed any concurrent medical or other psychiatric condition or substance disorder at the time of the assessment. This group consisted of patients who had been diagnosed at an interval of 6 months from the first episode psychosis (i.e. at the time of their presentation to the services). Diagnoses for these patients included schizophrenia, schizoaffective disorder and schizophreniform disorder, and diagnoses were made by experienced clinicians according to the DSM-5 criteria (American Psychiatric Association, 2013). The clinical assessment of the symptoms was performed using the Italian version of the Brief Psychiatric Rating Scale–24 (BPRS-24; Roncone et al., 1999). Each symptom on the 24-item scale was rated from 1 to 7 (1 = absence of symptoms; 7 = very severe symptoms).

Details about socio-demographic and clinical data of all participants are reported in Table 1. No linguistic, cultural or racial differences were present. We included only individuals who scored above the 25th percentile on Raven Progressive Matrices (Raven, 1938) or who had an Intellectual Quotient (IQ) above 85. All the participants were native Italian speakers and gave informed consent to participate. The data were collected from January 2014 to December 2019.

**Table 1:** Demographic and clinical information

	TD (N = 63)	ASD (N = 44)	SSD (N = 59)	F (df)	p
*Demographical information*					
Mean chronological age in years (sd)	21.4 (2.92)	20.8 (2.44)	21.9 (2.31)	2.51 (2,163)	.084
Mean education in years (sd)	13.5 (.50)	12.9 (1.85)	13.4 (2.76)	1.36 (2, 163)	.260
Gender (M; F)	42; 21	36; 8	43; 16	3.00 (2)^a^	.222
*Clinical information*					
*BPRS mean scores (St. dev.)*					
Total	–	–	52.3 (15.2)		
Positive symptoms	–	–	7.40 (3.61)		
Negative symptoms	–	–	6.67 (2.98)		
Depression	–	–	5.87 (2.60)		
Psychotic disintegration	–	–	21.0 (7.70)		
*ADOS-2 mean scores (St. dev.)*					
Total	–	14.5 (4.96)	–		
Social communication and social interaction	–	13.2 (4.81)	–		
Repetitive and stereotyped behaviours	–	1.32 (.707)	–		

^a^Chi-square test

### Instruments

#### ToM Measure

The Advanced Theory of Mind Task (A-ToM; Blair & Cipolotti, 2000) is an adaptation of Strange Stories, a ToM task first proposed by Happé (1994). It consists of 13 stories that describe real events. For a correct interpretation, the task requires the subject to go beyond the literal meaning of the text and make an inference about the story protagonist’s mental state. The 13 stories were designed to have only one possible interpretation, without ambiguity. Each story represents a different type of mental state attribution, i.e. Pretend (2 stories), Persuade, Joke, Lie (2 stories), White Lie (2 stories), Misunderstanding (2 stories), Irony, Double Bluff and Sarcasm.

Each story is followed by two questions: a comprehension question (e.g. ‘Was it true, what X said?’) and a justification question (e.g. ‘Why did X say that?’). For each item, a score of 1 is assigned when comprehension and justification questions are answered correctly; otherwise, a score of 0 is assigned. Thus, the total score ranges between 0 and 13. Happé (1994) used the term ‘advanced’ to refer to the two-level investigation of the story protagonists’ mental states to explain the cause of his/her behaviour (Pino & Mazza, 2016). The 13 A-ToM stories are described in the Online Appendix.

#### Classification of Responses and Scoring

With regard to the scoring phase, in this work we propose a new encoding of the 13 stories, in order to better discriminate between the styles of reasoning hidden in the mentalisation processes of ASD and SSD individuals. First of all, we allowed scoring only for responses to stories whose comprehension question was answered correctly. Subsequently, we assigned to each story one of the following three score levels:

- 0 = incorrect response for justification questions;
- 1 = the participant uses a reasoning style based on a physical state;
- 2 = the participant uses a reasoning style based on a mental state.

The incorrect response category (score = 0) applies to answers where: (a) the participant misunderstands the story situation; (b) inference is inappropriate with regard to the story protagonist’s utterance; and (c) the response reports the exact words used in the story.

The physical state category (score = 1) applies to answers referring to non-mental events, such as physical appearance, the action of an object, physical events and outcomes.

The mental state category (score = 2) applies to: (a) answers referring to thoughts, feelings, desires, traits and dispositions; and (b) answers where a figure of speech, used by the protagonist of the story, is correctly identified (e.g. justification answer: ‘It is a metaphor’). The rationale for this relies on evidence that ToM is strictly connected to pragmatic skills (Fernández, 2013; Frank, 2018; Resches & Pereira, 2007; for a more detailed description of these categories refer to Happé, 1994).

Thus, we classified as an incorrect response (score = 0) all those answers where an incorrect mental or physical attribution has been made.

An example of an A-ToM story (Joke) is the following: ‘*Giovanni, seeing the big dog of Chiara, exclaims: “Chiara, you don’t have a dog at all. You have an elephant*!”’ The referent justification question is: ‘*Why did Giovanni say that?*’ correct Mental state answers are: ‘*He made a joke*’ or ‘*It is just an expression people use*’ (score = 2). Correct Physical state answers are: ‘*Because the dog is big*’ or ‘*To indicate the dog’s size*’ (score = 1). Incorrect response answers are: ‘*Because it is a dog*’ or ‘*Because Giovanni made a mistake*’ (score = 0).

The frequencies of questions correctly comprehended by participants in each group are reported in Table 2.

**Table 2:** Frequencies of participants correctly addressing the comprehension questions of each A-ToM story

	Comprehension		p	Pairwise comparisons (p)		
	*No*	*Yes*		TD-ASD	TD-SSD	ASD-SSD
1_PRETEND						
TD	11 (17.5%)	52 (82.5%)	.280	–	–	–
ASD	3 (6.8%)	41 (93.2%)				
SSD	7 (11.9%)	52 (88.1%)				
13_PRETEND						
TD	3 (4.8%)	60 (95.2%)	.353	–	–	–
ASD	3 (6.8%)	41 (93.2%)				
SSD	7 (11.8%)	52 (88.2%)				
2_PERSUASION						
TD	5 (7.9%)	58 (92.1%)	** < .001**	.076	** < .001**	** < .001**
ASD	0 (0.0%)	44 (100.0%)				
SSD	21 (35.5%)	38 (64.5%)				
3_IRONIC_JOKE						
TD	5 (7.9%)	58 (92.1%)	**.001**	.339	**.007**	.144
ASD	6 (13.6%)	38 (86.4%)				
SSD	16 (27.1%)	43 (72.9%)				
10_IRONIC_JOKE						
TD	6 (9.5%)	57 (90.5%)	** < .001**	**.041**	**.029**	** < .001**
ASD	0 (0.0%)	44 (100.0%)				
SSD	15 (25.4%)	44 (74.6%)				
4_LIE						
TD	0 (0.0%)	63 (100%)	**.001**	1	.051	.133
ASD	0 (0.0%)	44 (100%)				
SSD	4 (6.7%)	55 (93.2%)				
5_LIE						
TD	1 (1.6%)	62 (98.4%)	** < .001**	1	**.028**	**.019**
ASD	0 (0.0%)	44 (100%)				
SSD	7 (11.8%)	52 (88.2%)				
6_WHITE_LIE						
TD	1 (1.6%)	62 (98.4%)	** < .001**	1	**.028**	**.019**
ASD	0 (0.0%)	44 (100%)				
SSD	7 (11.8%)	52 (88.2%)				
7_WHITE_LIE						
TD	0 (0.0%)	63 (100%)	** < .001**	1	**.002**	**.010**
ASD	0 (0.0%)	44 (100%)				
SSD	8 (13.6%)	51 (86.4%)				
8_MISUNDERSTANDING						
TD	3 (4.8%)	60 (95.2%)	**.001**	** < .001**	**.006**	.492
ASD	13 (29.5%)	31 (70.5%)				
SSD	13 (22.0%)	46 (78.0%)				
9_MISUNDERSTANDING						
TD	0 (0.0%)	63 (100%)	** < .001**	.066	**.001**	.227
ASD	3 (6.8%)	41 (93.2%)				
SSD	9 (15.2%)	50 (84.8%)				
11_DOUBLE_BLUFF						
TD	3 (4.8%)	60 (95.2%)	** < .001**	.087	** < .001**	**.042**
ASD	7 (15.9%)	37 (84.1%)				
SSD	21 (35.5%)	38 (64.5%)				
12_SARCASM						
TD	7 (11.1%)	56 (88.9%)	**.008**	**.039**	**.043**	**.004**
ASD	0 (0.0%)	44 (100.0%)				
SSD	10 (16.9%)	49 (83.1%)				

Significant results (p < .05) are reported in bold

### Procedure

Demographic data were obtained from each participant before the administration of A-ToM.

A-ToM was administered to each participant individually by a professional psychologist in a quiet room without any distractions; participants’ responses were registered using paper and pencil.

Each participant was instructed by the psychologist to answer the questions by trying to *put himself in the shoes* of the protagonist of the story. Moreover, the psychologist was present in the room during the administration to provide any information necessary for carrying out the task.

After the administration of the task, each justification question was categorised by six professional psychologists individually according to the three categories described above (mental state, physical state and incorrect response); afterwards, the overall concordance among raters was calculated (Fleiss K = 0.77; p < .001, showing high agreement among raters), and any disagreement was discussed until an agreement was reached.

The study was designed and conducted according to the principles established by the Declaration of Helsinki and informed consent was obtained from each participant before the test was administered.

### Data Analysis

A multinomial logistic regression model was performed, with A-ToM stories’ scores as independent variables and study group (TD, ASD and SSD) as the dependent variable, to estimate how likely stories are to predict the reasoning style. We used TD as the baseline in order to explain how A-ToM stories predict the reasoning style of ASD or SSD. We considered the three levels of response (0, 1, 2) as the modalities of an ordered discrete variable (progressing from the least to the most correct response, i.e. 0 to 1 to 2) so that the model prediction could reflect the correctness of the responses. Thus, regression beta coefficients indicate an increase or decrease in the risk of diagnosis based on the correctness of the response. A multinomial logistic regression model was performed as an omnibus test to understand which stories are suitable to significantly differentiate between typical and clinical populations (ASD or SSD) based on response style. In fact, if a story had significant results, it indicates that response style (incorrect, physical or mentalistic) is crucial for classifying the individual in one group or another; that is, how the participant responds to the story increases or decreases the relative odds of being in one of the clinical groups rather than in the typical group, showing a valid diagnostic performance, thus indicating that the style of response to that story could aid discrimination between groups. Between-group differences were further evaluated through contingency tables. In fact, we performed a chi-square analysis (or extended Fisher’s exact test as needed) of contingency tables for each of the 13 A-ToM stories, to verify the association between A-ToM scores and groups and to provide a better description of the differences. Moreover, a direct comparison between ASD and SSD was performed in order to describe differences between the two clinical group. Single stories are reported by story number followed by the story’s category; for example, A-ToM item 1, which represents the ‘Pretend’ category, is reported as 1_PRETEND. The analysis was performed using SPSS.

## Results

Multinomial logistic regression was performed to predict the reasoning style based on each of the A-ToM stories. Adding the predictors to a model that contained only the intercept significantly improved the fit between model and data (χ^2^ = 87.02, df = 26, Pseudo R^2^ = .569, p < .001).

Six stories resulted significantly for ASD, namely: 1_PRETEND (β = − 2.90, SE = 1.03, z = − 2.79, p = .005), 2_PERSUASION (β = − 2.64, SE = .966, z = − 2.73, p = .006), 4_LIE (β = − 2.70, SE = 1.26, z = − 2.14, p = .033), 7_WHITE_LIE (β = − 2.74, SE = .866, z = − 3.17, p = .002), 10_IRONIC_JOKE (β = − 2.68, SE = 1.14, z = − 2.33, p = .020) and 13_PRETEND (β = − .887, SE = .334, z = − 2.65, p = .008).

Five stories resulted significantly for SSD, namely: 1_PRETEND (β = − 4.05, SE = 1.32, z = − 3.06, p = .002), 2_PERSUASION (β = − 4.20, SE = 1.18, z = − 3.56, p < .001), 5_LIE (β = − 1.98, SE = .997, z = − 1.99, p = .047), 11_DOUBLE_BLUFF (β = − 1.32, SE = .459, z = − 2.89, p = .004) and 13_PRETEND (β = − .949, SE = .327, z = − 2.90, p = .004). The results for each story are reported in Table 3.

**Table 3:** Multinomial logistic regression: A-ToM stories as predictors of diagnosis of ASD or SSD, using TD as the baseline

	ASD		SSD	
	B	SE	B	SE
1_PRETEND	− 2.90*	1.03	− 4.05*	1.32
13_PRETEND	− .887*	.334	− .949*	.327
2_PERSUASION	− 2.64*	.966	− 4.20*	1.18
3_IROCNIC_JOKE	− 1.60	1.16	− 2.56	2.03
10_IRONIC_JOKE	− 2.68*	1.14	− 1.90	1.54
4_LIE	− 2.70*	1.26	.692	1.41
5_LIE	− 1.55	.904	− 1.98*	.997
6_WHITE_LIE	2.24	5.50	− 5.11	3.41
7_WHITE_LIE	− 2.74*	.866	− 1.35	1.06
8_MISUNDERSTANDING	.948	.854	− .772	.715
9_MISUNDERSTANDING	− 1.22	.699	− 1.23	.847
11_DOUBLE_BLUFF	− .184	.412	− 1.32*	.459
12_SARCASM	− 1.55	1.09	.043	1.15

*Significant predictor at p < .05

Frequencies and percentages of responses for each group of significant stories are reported in contingency tables in Table 4. Results in Table 4 confirmed those obtained through multinomial logistic regression.

**Table 4:** Response style for statistically significant A-TOM stories, only participants who passed the comprehension question were considered in the analyses

	Incorrect response	Physical state	Mental state	χ^2^ (df)	P
1_PRETEND					
TD	0 (0.0%)	17 (32.7%)	35 (67.3%)	–	< .001^a^
ASD	12 (29.3%)	8 (19.5%)	21 (51.2%)		
SSD	29 (55.8%)	5 (9.6%)	18 (34.6%)		
13_PRETEND					
TD	1 (1.7%)	–	59 (98.3%)		
ASD	7 (17.1%)	–	34 (82.9%)	–	< .001^ab^
SSD	21 (40.4%)	–	31 (59.6%)		
2_PERSUASION					
TD	5 (8.6%)	11 (19.0%)	42 (72.4%)	20.4 (4)	< .001
ASD	11 (25.0%)	10 (22.7%)	23 (52.3%)		
SSD	16 (42.0%)	11 (29.0%)	11 (29.0%)		
4_LIE					
TD	0 (0.0%)	34 (54.0%)	29 (46.0%)	–	< .001^a^
ASD	3 (6.8%)	26 (59.1%)	15 (34.1%)		
SSD	13 (23.6%)	26 (47.3%)	16 (29.1%)		
5_LIE					
TD	1 (1.6%)	20 (32.3%)	41 (66.1%)	–	< .001^a^
ASD	10 (22.7%)	23 (52.3%)	11 (25.0%)		
SSD	16 (30.8%)	17 (32.7%)	19 (36.5%)		
7_WHITE_LIE					
TD	1 (1.6%)	–	62 (98.4%)	–	< .001^ab^
ASD	15 (34.1%)	–	29 (65.9%)		
SSD	16 (31.4%)	–	35 (68.6%)		
10_IRONIC_JOKE					
TD	1 (1.7%)	18 (31.6%)	38 (66.7%)	–	< .001^a^
ASD	2 (5.0%)	16 (40.0%)	22 (55.0%)		
SSD	18 (40.9%)	12 (27.3%)	14 (31.8%)		
11_DOUBLE_BLUFF					
TD	0 (0.0%)	16 (26.7%)	44 (73.3%)	–	.010^a^
ASD	2 (5.4%)	9 (24.3%)	26 (70.3%)		
SSD	3 (15.8%)	9 (47.4%)	7 (36.8%)		

^a^Fisher-Freeman-Hamilton exact test

^b^Applied to a 2 × 3 table

Comparing frequencies of A-ToM scores (0, 1, 2; i.e. incorrect, physical or mental response) between ASD and SSD, we found significant results regarding 1_PRETEND (χ^2^ = 6.76, df = 2, p = .038), 3_IRONIC_JOKE (χ^2^ = 16.44, df = 2, p < .001), 6_WHITE_LIE (p = .001, FFH exact test), 9_MISUNDERSTANDING (p = .005, FFH exact test), 10_IRONIC_JOKE (χ^2^ = 14.99, df = 2, p = .001), 11_DOUBLE_BLUFF (p = .042, FFH exact test) and 13_PRETEND (χ^2^ = 5.92, df = 1, p = .015). Frequencies and percentages of significant stories comparing ASD and SSD responses are reported in Table 5.

**Table 5:** Response style for significant A-TOM stories in clinical groups (ASD, SSD), only participants who passed the comprehension question were considered in the analyses

	Incorrect response	Physical state	Mental state	χ^2^ (df)	p
1_PRETEND					
ASD	12 (29.3%)	8 (19.5%)	21 (51.2%)	6.76 (2)	.038
SSD	29 (55.8%)	5 (9.6%)	18 (34.6%)		
13_PRETEND					
ASD	7 (17.1%)	–	34 (82.9%)	5.92 (1)^b^	.015
SSD	21 (40.4%)	–	31 (59.6%)		
3_IROCNIC_JOKE					
ASD	0 (0.0%)	30 (78.9%)	8 (21.1%)	16.44 (2)	< .001
SSD	14 (32.6%)	19 (44.2%)	10 (23.2%)		
10_IRONIC_JOKE					
ASD	2 (5.0%)	16 (40.0%)	22 (55.0%)	14.99 (2)	.001
SSD	18 (40.9%)	12 (27.3%)	14 (31.8%)		
6_WHITE_LIE					
ASD	4 (9.1%)	3 (6.8%)	37 (84.1%)	–	.001^a^
SSD	20 (38.5%)	1 (1.9%)	31 (59.6%)		
9_MISUNDERSTANDING					
ASD	17 (41.5%)	0 (0.0%)	24 (58.5%)	–	.005^a^
SSD	27 (54.0%)	7 (14.0%)	16 (32.0%)		
11_DOUBLE_BLUFF					
ASD	2 (5.4%)	9 (24.3%)	26 (70.3%)	–	.042^a^
SSD	3 (15.8%)	9 (47.4%)	7 (36.8%)		

^a^Fisher-Freeman-Hamilton exact test

^b^Applied to a 2 × 2 table

## Discussion

The present study aimed to evaluate mentalising ability using a ToM test in a clinical setting in ASD and SSD subjects, compared to TD subjects.

It also had the objective of examining whether the style in the attribution of mental states, even if compromised in both disorders, presented qualitative differences between ASD and SSD.

An Italian adaptation of Strange Stories, the A-ToM (Blair & Cipolotti, 2000; Prior et al., 2003), was used. The Strange Stories task is a widely used test and is described in the literature as an advanced measure of the ability to infer mental states in socially complex scenarios (Livingston et al., 2019). Moreover, many authors have proposed modified versions of the ‘classic’ set of Strange Stories (Brewer et al., 2017; Dziobek et al., 2006; Murray et al., 2017).

We used the A-ToM to analyse the reasoning style hidden in the process of mentalisation when solving ToM tasks. Understanding the reasoning style individuals use to interpret other people’s mental state could help clinicians to discriminate between the typical answers of ASD and SSD subjects. Persons with SSD or ASD share the same ToM disorder (Barneveld et al., 2014; Baron-Cohen et al., 2015; Maat et al., 2012; Martinez et al., 2019). Exploring the different manifestations of ToM could lead to understanding whether and how this domain can be considered a marker of these disorders. For example, Hoogenhout and Malcolm-Smith (2017) found that ToM skills could identify the severity of ASD in children. To date, few studies (Martinez et al., 2019; Sasson et al., 2011) have compared young adults with ASD and SSD during the diagnostic process in order to understand what type of reasoning each subject uses to identify the mental states of other people. Other studies have evaluated the style of response of SSD individuals in ToM tasks: for example, Fretland et al. (2015) found that an over-mentalising style of responses was associated with positive symptoms in SSD and that overly simplistic answers could be due to a lack of accuracy in the style of responses rather than a complete lack of mentalising abilities. Scherzer et al. (2012) found that in the Strange Stories task SSD responses were characterised by non-interpretative and incorrect attributions rather than by incomplete attributions.

Using TD as a baseline, our results showed that six ToM stories are likely to predict a typical response style of ASD compared to TD, while five ToM stories are likely to predict a typical response style of SSD compared to TD. The SSD group showed greater difficulty in understanding social scenarios and could not solve the comprehension questions. On the other hand, the ASD group understood the stories, but did not correctly identify the intention of the protagonist, thereby showing that ASD participants are characterised by a pure mentalising difficulty.

### Pretend

Both clinical groups had difficulty responding to stories that investigate the construct of *Pretend*, as compared to the TD group. *Pretend* is the ability to create imaginary events and to establish alternative identities for objects and people (Bruner, 1972; Rutherford & Rogers, 2003). According to Leslie (1987), pretend play is an early manifestation of the meta-representational abilities necessary for the development of a ToM. As suggested by Lillard (1993), pretend play is based on the ability to hold two mental representations in the mind (Kang et al., 2016; Leslie, 1987; Lillard, 1993). While the first representation reflects the state of the real world (perceived situation), the second reflects the pretend situation (Kang et al., 2016). Our results showed that participants in the SSD group were significantly more likely to respond incorrectly to both pretend stories (1_PRETEND and 13_PRETEND) than participants in the ASD group. Specifically, participants with SSD showed a tendency to attribute bad intentions to characters who are playing pretend or distorting reality (e.g. *Emma is a liar; Emma teases Katie; She doesn’t want to give the banana to Katie; Philip is wrong*). This result could be related to the incapacity of SSD individuals to use contextual information to make appropriate inferences about other people’s mental states (Frith, 1992; Langdon et al., 2001).

On the other hand, even when ASD participants answer the justification question incorrectly, they remain anchored to the specific context (e.g. *Children don’t have much imagination; In fantasy everything is possible; Philip says it just for fun*). In addition, our results highlighted a higher probability that the ASD group would respond using a mentalistic style in their responses than the SSD group (e.g. *She’s pretending that banana is a phone; Philip pretends to be a pirate*). According to the literature, most children with ASD exhibit an impairment of the ability to *‘play pretend’* (Gallagher & Varga, 2015; Happé, 1995; Happé & Frith, 2014; Jarrold, 2003; Kang et al., 2016; Leslie, 1991). However, it seems that many individuals with ASD may have an underlying ability to understand others’ actions of pretend, even if they fail to engage in spontaneous pretend (Jarrold, 2003, Jarrold et al., 1994; Kang et al., 2016; Kavanaugh & Harris, 1994).

### Persuasion

Our clinical groups had significant difficulty, as compared to the TD group in responding to stories describing *Persuasion.* This construct requires understanding of a symbolic process that involves an attempt to bring about a change in attitudes, or to instil a particular belief in another person in an atmosphere of free choice (Lonigro et al., 2017; Perloff, 2010; Petty & Briñol, 2015). The central core of persuasion is the understanding that other people have different mental states that can be changed (Lonigro et al., 2017). This process requires adequate ToM skills. Interestingly, this *once again* underlines that SSD individuals tend to attribute bad intentions to the protagonist of the story. For instance, in regard to 2_PERSUASION, individuals with SSD are more likely to believe that the woman intended to drown the kittens, thereby attributing negative intentions to her. This paranoid ideation reflects the tendency of some individuals with schizophrenia to over-attribute intention to others (Green et al., 2015; Scherzer et al., 2012); negative symptoms and some of the positive symptoms of SSD, like paranoia, can be explained as impairment in the initiation and monitoring of willed actions. This reflects a difficulty in knowing whether or not an action was one’s own.

Incorrect responses from participants in the ASD group showed an inability to integrate all information to achieve a whole meaning (e.g. *Nobody wanted to take the kittens; Mrs Rossi can’t keep all the kittens; Mrs. Rossi is forced to abandon the kittens; She cares a lot about her kittens*). We support the idea that 2_PERSUASION, which involves manipulation, strategic deception and feelings of guilt, requires is too complex for individuals with autism, as it requires that participants adopt multiple social perspectives.

### Double Bluff

Our attention was focused on 11_DOUBLE_BLUFF: it is the only story that evaluates third-order ToM (Happé, 1994). In third-order ToM, the meta-representational level of recursivity improves until the third embedded belief (e.g. ‘I think that you think that he/she thinks that another person thinks…’ Happé, 1994; Valle et al., 2015). According to Valle et al. (2015), the third-order false belief task requires you to predict the character’s behaviour after attributing to him a mental state. The complexity of the recursive thinking of the third-order ToM can directly detect the implicit meta-representative mechanisms necessary to attribute a mental state and predict behaviour based on attribution. It is believed that ToM abilities continue to develop during adolescence and, in particular, during early adulthood, when higher-order recursive thinking emerges (Valle et al., 2015). Several studies have investigated how the development of ToM is linked to cognitive skills such as language, memory and executive functions; this relationship has been found in both clinical populations (SSD and ASD) and the TD population (Apperly et al., 2009; Demetriou et al., 2018; German & Hehman, 2006; Leung et al., 2016; Marinopoulou et al., 2016; Mutter et al., 2006; Valle et al., 2015; Wang et al., 2018).

Nonetheless, it seems that accuracy in the use of recursive thinking is closely related to social experience and the ability to predict the behaviour of others (Valle et al., 2015). As highlighted above, psychotic disorders mostly emerge during adolescence and early adulthood, and this coincides, therefore, with the development of higher-order ToM skills. According to Wang et al. (2018), it is not surprising that there is an impairment of ToM in psychotic patients (Mazza et al., 2010). In the 11_DOUBLE_BLUFF, one-third of SSD participants miss the comprehension question because the social scenario is too complex (see Table 2).

The SSD group shows a tendency to miss the justification question in all the constructs. Instead, the ASD group shows better performance by using mostly mental reasoning. This trend is not confirmed for the story that investigates the joke construct, where the ASD group shows a tendency to use physical reasoning. Verbal irony is a form of figurative or non-literal language in which the intended meaning of the speaker is frequently different from the literal meaning of his or her words (Pexman et al., 2011). Understanding ironic jokes (Sullivan et al., 1995) involves complex inferences about the speaker’s mind, requires attention to several social cues and is related to the ability to attribute at least second-order mental states (Deliens et al., 2018; Pexman, 2008). In stories involving a joke, the speaker knew that the listener knew the truth (a true second-order belief) and did not expect the listener to believe what was said. The literature has shown that individuals with ASD have deficits in the interpretative skills required for figurative language comprehension, compared to their TD peers (German & Hehman, 2006; Happé, 1994; Losh & Capps, 2006). Moreover, when presented with an ironic scenario, the ASD person tends to use explanations that involve reinterpreting the context to make the literal meaning fit (MacKay & Shaw, 2004; Pexman et al., 2011).

### Ironic Joke

Irony is a type of figurative language that is used to convey messages in an indirect and sometimes amusing way (Saban-Bezalel et al., 2019). Irony is widely used in everyday life (Gibbs, 2000), and it helps to strengthen social bonds (Clark & Gerrig, 1984; Saban-Bezalel et al., 2019). Understanding irony requires the ability to go beyond literal language through complex mental representations (Saban-Bezalel et al., 2019). We found that our clinical groups had difficulty responding to stories that investigate irony. In the 3_IRONIC_JOKE, where the protagonist calls a dog an elephant, about four out of five ASD participants use a physical justification, for example, ‘the dog is big like an elephant’ or ‘because Giovanni sees a dog as big as an elephant’, evidencing little understanding of the mental states involved (ironic joke). This is in line with the literature, which highlights how ASD individuals perform better in metaphorical tasks, where the ability to accomplish first-order ToM tasks (inferring a person’s mental state, e.g. what he/she thinks) is required and a literal interpretation is sufficient (Happé, 1994; Sperber & Wilson, 1986). As for the SSD group, they showed high percentages of incorrect responses. In both ironic joke stories at least one out of three did not provide a correct mental or physical response to the justification question; examples of incorrect responses included *‘Because Giovanni was an elephant’, ‘Because he had this feeling*’, for 3_IRONIC_JOKE, and *‘Hair is cut short to make other people laugh’, ‘to keep the grass from being mown’* for 10_IRONIC_JOKE (see Online Appendix). The qualitative analysis of their answers shows that they tend to use language characterised by reduced semantic coherence and syntactic complexity as well as by reduced vocabulary (Andreasen & Grove, 1986; Corcoran et al., 2018). Previous studies concerning SSD have reported that ToM difficulties are associated with dysfunctions in the processing of visual-perceptual and linguistic context (Dwyer et al., 2019; Schenkel et al., 2005), but they do not clarify the specific modes of attribution of mental states that the subjects use to understand and predict the behaviour of others.

## Limitations

We have to acknowledge the limitations of our study. Firstly, in our study we used a cognitive ToM test, but it is worth pointing out that there are several theoretical models of ToM (e.g. affective and cognitive ToM; Shamay-Tsoory & Aharon-Peretz, 2007).

Another limitation concerns the TD group. It is not very convincing that the TD group makes so many mistakes to some ToM stories, such as 1_PRETEND, 10_IRONIC_JOKE and 12_SARCASM. We think this is a problem not to be underestimated in the field of psychology when involving control groups, and is perhaps due to low motivation to take the test or to control group participants’ underestimation of the test itself because they think the test is very simple and have a superficial attitude. In addition, we did not use a clinical assessment for this group, but during ToM test administration, TD participants had to answer a questionnaire for socio-demographic data where they were asked whether they had a history of neurological disease, including epilepsy, psychiatric disorders, substance disorders and head trauma. All participants in the TD group claimed not to have received any clinical diagnosis.

The lack of objective measures to assess language and emotional vocabulary skills is another limitation of our study; for this reason, we suggest that cognitive abilities (e.g. executive functions, adaptive skills, language skills) should also be evaluated during ToM assessment.

Finally, in our study we did not consider the important problem of comorbidity in the SSD and ASD sample. For example, we did not consider catatonia as comorbidity in both clinical conditions. According to Dhossche et al. (2015), catatonia is a comorbid syndrome occurring in 12–17% of selected groups of adolescents and young adults with autism spectrum disorders who have been referred for specialised care or admitted to hospital. In fact, in clinical practice it is common to misinterpret catatonic symptoms, including mutism, stereotypic speech, repetitive behaviours, echolalia, posturing, mannerisms, purposeless agitation and rigidity, as features of ASDs (Mazzone et al., 2014). There may be many similarities between ASD and catatonia, but definitive conclusions need to be deferred until future research is conducted with designs allowing direct comparisons of patients with autism and catatonia.

## Conclusion

In conclusion, our study underlines the difficulty of making a differential diagnosis between SSD and ASD. It’s important to emphasise that ToM is generally recognised as an important treatment target for both clinical groups (Fernanes et al., 2018; Kuo et al., 2019; Mazza et al., 2010; Morrison et al., 2019; Pinkham et al., 2016, 2018), and therefore the use of well-validated measures capable of distinguishing between the two conditions seems to be crucial.

As routine clinical and cognitive tests do not always identify the main characteristics of the two disorders (Pino et al., 2020), we have proposed in our paper a version of a ToM test, useful for understanding the differences between a person with ASD and a person with SSD in mentalising ability, which is impaired in both disorders, but at different levels. Regarding SSD, our data seem to provide support for the identification of some response schemes as characteristic of SSD individuals while performing a ToM task. The difficulties in understanding ToM stories could be related to an inability to coordinate multiple pieces of information simultaneously (Dwyer et al., 2019; Frith, 1989; Schenkel et al., 2005; Silverstein & Phillips, 2003). In addition, the difficulties in understanding questions could be due to the complexity of the scenarios, which present advanced concepts such as double bluff, white lies and persuasion, and to the cognitive processes involved, for example working memory, verbal fluency and deductive reasoning (Ahmed et al. 2007; Ahmed and Miller 2011; Happé, 1994). The link between cognitive (non-social) functions and performance on ToM tasks among individuals with prodromal psychosis has been highlighted in the literature (Brune et al., 2003; Zhang et al., 2016).

Regarding ASD individuals, they understand the stories better than SSD individuals, but they do not correctly identify the intention of the protagonist, thereby showing that ASD participants are characterised by a pure mentalising difficulty.

Although further evidence is required, we believe that the use of A-ToM stories as a routine tool in a clinical setting could provide important information to clinicians, especially in young adult patients.

## Supplementary Information

Below is the link to the electronic supplementary material.

Supplementary file1 (DOCX 21 kb)
