# Supplementary material for: Qualitative Differences in Attribution of Mental States to Other People in Autism and Schizophrenia: What are the Tools for Differential Diagnosis?
Source: J Autism Dev Disord. 2021 Apr 28;52(3):1283–98. doi: 10.1007/s10803-021-05035-3 (PMC8854268; doi:10.1007/s10803-021-05035-3)
Supplement: Supplementary file 1 — Supplementary file1 (DOCX 21 kb) [file 10803_2021_5035_MOESM1_ESM.docx]

***APPENDIX***

***The main overlaps, regarding impairment in SC abilities, between ASD and SSD***

| **AUTISM SPECTRUM DISORDER** | **SCHIZOPHRENIA SPECTRUM DISORDER** |
| --- | --- |
| **Deficit in social communication and social interaction** | **Negative symptoms** |
| - Lack of socio-emotional reciprocity - Affective flattening - A deficit in non-verbal communication - Avoidance of eye contact - Reduction or lack of facial expression - A deficit in understanding and using emotional gestures - Difficulties in developing, maintaining and understanding relationships | - Poverty of speech - Affective flattening - Diminished emotional expression - Reduction of eye contact - Reduction of facial emotional expressions - Anomalies in prosodies - Anhedonia - Asociality and social withdrawal |

**The mental states investigated in the 13 A-ToM stories**

| **Mental State Attribution** | **Explanation of Mental State** | **A-ToM Stories** |
| --- | --- | --- |
| PRETEND | Capacity to represent absent objects and situations, “acting as if” (Leslie, 1987). | Story 1  Katie and Emma are playing in the house. Emma picks up a banana from the fruit bowl and holds it up to her ear. She says to Katie, **“Look! This banana is a telephone!”**  *Is it true what Emma says?*  *Why does Emma say this?*  Story 13  Marco and Filippo are having fun! They have overturned the table on the ground and are sitting on it. When their mother returns, she laughs and says, “What on earth are you doing?” **“This table is a pirate ship!”** says Philip, **“and you’d better get in before drowning because you’re in the sea!”**  *It is true what Filippo says?*  *Why does Filippo say this*? |
| PERSUASION | The symbolic process that involves an attempt to bring about a change in attitudes or instil a particular belief in another person in an atmosphere of free choice (Perloff, 2010; Petty & Brinol, 2015; Lonigro et al., 2017). | Story 2  Giuseppe wanted to buy a kitten, so he went to see Mrs Rossi, who had lots of kittens she didn’t want. Now Mrs Rossi loved the kittens, and she wouldn’t do anything to harm them, though she couldn’t keep them all herself. When Giuseppe visited he wasn’t sure he wanted one of Mrs Rossi’s kittens since they were all males and he had wanted a female. But Mrs Rossi said, **“If no one buys the kittens I'll just have to drown them!”**  *It is true what Mrs Rossi said? d Smith says this to Jane?*  *Why does Mrs Rossi say this to Giuseppe?* |
| IRONIC JOKE | In the joke/ironic stories, the speaker knew that the listener knew the truth (a true second-order belief) and did not expect the listener to believe what was said (Sullivan et al., 1995; Winner et al., 1998). Thus, the implicit communicative intent of the speaker is contradictory to what is explicitly expressed. To understand irony it is necessary to decode the social context and the intention of the speaker (Sperber & Wilson, 1995, 2002). | Story 3  Giovanni went to Chiara’s house for the first time. John arrived at Chiara’s house, she opened the door and her dog ran to greet Giovanni. Chiara’s dog is huge: it’s almost as big as Giovanni! When John saw Claire’s huge dog he said: **“Claire, you haven’t got a dog at all. You’ve got an elephant!”**  *Is it true what Giovanni said?*  *Why does Giovanni say this?*  Story 10  One day, Daniele and Luca see Mrs Verdi coming out of the hairdresser. She makes you laugh a bit because the hairdresser cut her hair too short. Daniele says to Luca: **“She must have been in a fight with a mower!”**  *It is true what Daniel said?*  *Why does Daniel say this?* |
| LIE | A lie is a deliberate form of deception consisting of three main elements: it communicates information; the person who communicates the message wants to mislead; the person who receives the message perceives the information as true (Ludwig, 1965; Stokke, 2013). | Story 4  One day, while playing at home, Anna accidentally knocked over and broke her mother’s favourite crystal vase. Anna knew that her mother would be very angry! So when the mother came home and saw the broken vase and asked Anna what had happened, Anna said, **“The dog knocked him over, it wasn’t my fault!”**  *It is true what Anna told her mother?*  *Why does Anna say this?*  Story 5  Giovanni hates going to the dentist because every time he goes he has to have a filling, which hurts him a lot. But Giovanni knows that when he has a toothache, his mother always takes him to the dentist. Now Giovanni has severe toothache, but when his mother notices that he is suffering and asks him, **“Do you have a toothache, Giovanni?”,** Giovanni replies: **“No, mom.”**  *It is true what Giovanni says to his mother?*  *Why did Giovanni say this?* |
| WHITE LIE | This is a lie that is said for the sake of good, to hide from the person concerned a painful truth (prosocial). | Story 6  Elena waited all year for Christmas to ask her parents for a bunny. Christmas day arrived and Elena ran to open her gift. She was sure it contained a small rabbit in a cage. But when she opened it, with all the family around, she discovered that her gift was a boring encyclopedia, which Elena did not want at all! Then, when Elena’s parents asked her how much she had liked her Christmas present, she said**: “It is beautiful, thank you. It is just what I wanted.”**  *It is true what Elena says?*  *Why did Elena say this to her parents?*  Story 7  One day Aunt Lucia came to visit Pietro. Usually, Pietro likes his aunt, but that day she wore a new hat that Pietro didn’t like. Pietro thought that his aunt looked ridiculous in that hat and that she would have been much better with the old one. But when Aunt Lucia asked Pietro: **“Do you like my new hat?”** Pietro replied: **“Oh, it’s very beautiful.”**  *Is it true what Pietro said?*  *Why did Pietro say it?* |
| MISUNDERSTANDING | This is an [occasion](https://dictionary.cambridge.org/dictionary/english/occasion) when someone does not [understand](https://dictionary.cambridge.org/dictionary/english/understand) something [correctly](https://dictionary.cambridge.org/dictionary/english/correctly). | Story 8  Late at night, old Mrs Bianchi is returning home. She does not like to walk home alone in the dark because she is always worried that someone will attack and rob her. Suddenly a man emerges from a shadow. He wants to ask Signora Bianchi what time it is, so he walks towards her. When Mrs Bianchi sees the man walking towards her, she starts to tremble and says: **“Take my bag, but don’t hurt me, please!”**  *Was the man surprised at what Mrs Bianchi said?*  *Why did she say this to him, since he just wanted to ask her what time it was?*  Story 9  A thief who had just stolen from a shop was running away. As he ran home, a policeman on duty saw him lose a glove. The policeman did not know that the man was a thief, and only wanted to tell him that he had lost a glove. But when the policeman shouted to the thief, “Hey, you! Stop!”, the thief turned, saw the policeman and surrendered. **With his hands up he admitted that he had committed the wrongdoing in the shop.**  *Will the policeman have been surprised at what the thief said?*  *Why did the thief behave like this, since the policeman just wanted to return his glove to him?* |
| DOUBLE BLUFF | A double bluff is a clever attempt to deceive someone by telling that person the truth when they think you are telling lies. This story type requires a third-order theory of mind (i.e. an extra level of embedding; a character does tell the truth but does so in the knowledge that his listeners will not believe him and will, therefore, be misled as he intends; Happé, 1994) | Story 11  Simone is a great liar and Massimo, Simone’s brother, knows that Simone never tells the truth! Just yesterday Simone stole Massimo’s ping-pong bat, and Massimo knows that Simone hid it somewhere, despite not being able to find it. He is very angry, so he says to Simone: “Where’s my ping-pong bat? You must have hidden it in the closet or under the bed because I’ve looked everywhere. Where is it, in the closet or under the bed?” **Simone replies that it is under the bed.**  *Will what Simone said to Massimo be true?*  *Where will Massimo look for his ping-pong bat?*  *Why will he look for his bat there?* |
| SARCASM | It is a form of verbal irony that aims to communicate an opposite attitude to the thought expressed by the sarcastic expression. The listener recognizes the gap between the semantic content of the ironic expression and the true communicative intent of the speaker (Wilson & Sperber, 2012). | Story 12  Anna’s mother spent a lot of time cooking Anna’s favourite dish: fish and chips. But when she takes it to Anna, she is watching TV, she doesn’t consider her and she doesn’t even thank her. Anna’s mother is angry and says, **“Well, that’s nice behaviour, isn’t it? This is pure rudeness to me!”**  *It is right what Anna’s mother said?*  *Why did Anna’s mother say this?* |
